# Biomedical and Clinical Promises of Human Pluripotent Stem Cells for Neurological Disorders

**DOI:** 10.1155/2013/656531

**Published:** 2013-09-22

**Authors:** Nopporn Jongkamonwiwat, Parinya Noisa

**Affiliations:** ^1^Faculty of Health Sciences, Srinakharinwirot University, Ongkharak, Nakhon Nayok 26120, Thailand; ^2^School of Biotechnology, Institute of Agricultural Technology, Suranaree University of Technology, Nakhon Ratchasima 30000, Thailand

## Abstract

Neurological disorders are characterized by the chronic and progressive loss of neuronal structures and functions. There is a variability of the onsets and causes of clinical manifestations. Cell therapy has brought a new concept to overcome brain diseases, but the advancement of this therapy is limited by the demands of specialized neurons. Human pluripotent stem cells (hPSCs) have been promised as a renewable resource for generating human neurons for both laboratory and clinical purposes. By the modulations of appropriate signalling pathways, desired neuron subtypes can be obtained, and induced pluripotent stem cells (iPSCs) provide genetically matched neurons for treating patients. These hPSC-derived neurons can also be used for disease modeling and drug screening. Since the most urgent problem today in transplantation is the lack of suitable donor organs and tissues, the derivation of neural progenitor cells from hPSCs has opened a new avenue for regenerative medicine. In this review, we summarize the recent reports that show how to generate neural derivatives from hPSCs, and discuss the current evidence of using these cells in animal studies. We also highlight the possibilities and concerns of translating these hPSC-derived neurons for biomedical and clinical uses in order to fight against neurological disorders.

## 1. Introduction

Neurological disorders include a variety of hereditary and sporadic diseases that involve the chronic and progressive loss of neuronal structures and functions. We can divide these into two major groups depending on the onset of disease, which are (1) early onset neurodevelopment diseases and (2) late onset neurodegenerative diseases. Since aging is the most consistent risk factor for neurodegenerative diseases and we are now experiencing an increase in the numbers of the elderly population, it is of great importance to develop the treatments for these diseases. Although the rapid development of novel diagnostic methods and therapeutic approaches are in the stages of development, there is limited evidence of effective systems that can prevent and cure the diseases. Cell replacement therapy by using stem cells is a promising strategy to treat these diseases because certain pathological conditions are affected by neuronal loss. The possibility of generating abundant differentiated cells from human stem cells for cell replacement therapies provides a plausible avenue to treat such diseases. Neural stem cells that are isolated from adult donor or from fetal brain tissues have been considered as reasonable resources for this purpose; however, adult stem cells are of a very limited quantity, and histocompatibility is a major drawback.

Human pluripotent stem cell (hPSC) technologies, including human embryonic stem cells (hESCs) and induced pluripotent stem cells (iPSCs), are potentially prominent processes for manipulating human illnesses in terms of disease modeling, tissue engineering, drug discovery, and cell therapy. The pluripotent developmental potential of hPSCs and the success of transplanting their differentiated derivatives in animal disease models demonstrated the principle of using hPSC-derived cells as a regenerative source for transplantation therapies for human diseases. However, before hPSCs can be translated into clinical use, a thorough understanding of the basis of hPSCs is mandatory. One of the major concerns that hinders the application of hPSCs for cell and tissue therapy in human is histocompatibility. Notably, recent data support the concept that hESCs and their differentiated derivatives possess immune-privileged properties [1], suggesting that cells derived from hESCs may provide a potential tool for the induction of immunotolerance [2]. In another scenario, for which the term “personalized pluripotent cells” has been coined, people could use their own somatic cells to be reprogrammed back to the pluripotent cell state [3]. The feasibility of reprogramming was first demonstrated by somatic cell nuclear transfer (SCNT) or cloning [4]. Somatic cells of patients are fused with enucleated oocytes; thereafter, hESCs could be established in culture and be induced to *in vitro* differentiation to provide patient-specific cells and tissues [5]. However, the reprogramming of the somatic nucleus in an oocyte is still inefficient. In addition, accessing a source of human oocytes is not only a rare opportunity but it is also the ethical concern of the moment [6]. As an alternative to reprogramming by SCNT, adult human fibroblasts can be directly reprogrammed into a state that is similar to that of hESCs by the expression of only four factors: OCT4, KLF4, SOX2, and c-Myc [7] and such reprogrammed cells are termed as “induced pluripotent stem cells” or iPSCs.

Since hPSCs have great potential to differentiate into all cell types, the most common strategy in salvaging the neural function after injuries is not the direct transplantation of these pluripotent cells but applying their various differentiated cells instead. Specification of hPSCs *in vitro* along neural progenitor pathways would also allow the investigation of early human brain development, including regulatory signals for cell commitment and neurogenesis. Additionally, the cells could be used for the screening for new drugs and carcinogenic or toxic compounds that cannot be analyzed *in vivo* due to limited samples and ethical constraints. Ultimately, the potential utility of hPSC-derived neurons for treating neurological diseases is just at the beginning because there are several issues that need to be taken into account, that is, efficiency, safety, and functionality. In this paper, we review the promising benefits of hPSCs for the analysis and treatment of neurological disorders.

## 2. Approaches for the Derivation of Neural Derivatives from Human Pluripotent Stem Cells

The transplantation of stem cells which are committed to a specific lineage can avoid *in vivo* teratoma formation which is caused by the rapid growth and uncontrolled spontaneous differentiation of hPSCs [8]. Nevertheless, an efficient differentiation of hPSCs into clinically appropriate progenitors or specific targeting cell types remains a key challenge. The differentiation studies of hPSCs have intensively focused on exploring the roles of growth factors and small molecules [9–12]. Several approaches have been used to achieve *in vitro* neural differentiation from hPSCs, aimed at generating regionally specified neural progenitors or differentiated neurons/glial subtypes [13]. Reportedly, hPSCs have been differentiated successfully into several types of neural derivatives, including neural progenitor cells [11, 12, 14–20], neural crest progenitors [21], motor neurons [22–25], sensory neurons [9], dopaminergic neurons [26, 27], and specific glial subtypes [28]. The differentiation systems were initially achieved through cell aggregation or embryoid body (EB) formation in the presence of retinoic acid (RA) as a starting point for the isolation and culture of highly purified populations of neural progenitor cells [16]. These progenitors were able to be cultivated for about 25 population doublings as neurospheres in suspension culture, and they express markers of the early neuroectoderm, such as Nestin, polysialylated (PSA) NCAM, Musashi1, and PAX6 [16]. The neural progenitor cells were able to be differentiated into neurons, astrocytes, and to a minor degree of cells expressing oligodendrocyte markers. However, as hPSCs are pluripotent, the efficiency of neural conversion is limited and lineage selection is usually needed to ensure the enrichment of a specific differentiated population. Most of the lineage induction protocols employed the addition of morphogens or growth factors to the hESC aggregates in suspension cultures. For this reason, EB formation has some drawbacks. Because a high concentration of morphogens or growth factors is required in order for the factors to reach cells inside the aggregates [29], cells on the surface and those inside the aggregates will present a varied degree of exposure to morphogens, which create a wide range of cell lineages or cells at various developmental stages. In addition, it is impossible to visualize the continual change of cell morphology in response to treatment because of the cluster nature.

To overcome these drawbacks, a simpler way to reconstitute neural commitment *in vitro* and achieve efficient neural production relies upon monolayer differentiation of hPSCs. However, when applying a similar monolayer differentiation system used for directing mouse embryonic stem cells (mESCs) to neural lineage, hPSCs generated a large proportion of nonneural lineage cells. This is mainly due to the active BMP signaling pathway in hPSCs [30]. Therefore, the only approach that has been shown to induce efficient hESC neural differentiation is by directly inhibiting the BMP and/or SMAD signaling pathways [12, 31, 32]. Treatment of hPSCs with noggin, a BMP antagonist, generated a homogeneous, morphologically distinct population of cells that expressed neuroectodermal markers, including PAX6, Musashi1, and SOX2 and with no detection of mesoderm and endoderm lineage markers [12]. Noggin alone appears to, at least, initiate hPSCs differentiation toward neural lineage. The formation of “neural rosettes” is another morphological marker of *in vitro *differentiation of hESCs to neural cells which is reminiscent of the *in vivo* structural formation of a developing neural tube [33]. The culture of hPSCs in chemically defined medium with noggin resulted in PAX6^+^/SOX1^−^ neural rosettes, and the additional supplementation of fibroblast growth factor 2 (FGF2) induced PAX6^+^/SOX1^+^ neural rosettes [34]. Rosette-forming neural stem cells expressing anterior markers of the nervous system, such as Forse1, have shown the broadest differentiation potential [35]. These cells propagated in the presence of FGF2 and retained Forse1 expression, even though FGF2 was considered to caudalize the cell fate of neural stem cells [36]. Moreover, the cells in neural rosettes are capable of multiplying by symmetrical division over a period of time and are able to differentiate cell types of both anterior-posterior and central-peripheral types of the nervous system and could be maintained in a long-term culture by stimulating Sonic hedgehog (Shh) and Notch pathways [35]. Neural progenitor cells derived from hPSCs are also capable of producing astrocytes and oligodendrocytes, either under basal conditions or with the medium supplemented with ciliary neurotrophic factor or platelet-derived growth factor (PDGF) [37]. Mature astrocytes express specific astroglial markers such as glial fibrillary acidic protein (GFAP) and S100*β*. However, the proportion of oligodendrocytes differentiating from hPSCs among the differentiated progeny is generally very low [17]. It has been shown that Olig2^+^ neural progenitors can be readily differentiated from hESCs in response to SHH and RA [38]. These Olig2^+^ progenitors generate mostly motor neurons during the neurogenic period. Moreover, Olig2^+^ progenitors persist after neurogenesis and become oligodendrocytes. This suggests that the Olig2^+^ progenitors may differentiate into oligodendrocytes, and highlights the importance of Olig2 in oligodendrocyte development [39]. It is noted that during embryonic neurodevelopment, glial cells, for example, astrocytes and oligodendrocytes are generated after the birth of major neuronal types [40]. The same neurogenesis to gliogenesis transition is preserved when neuroectodermal cells are cultured [41] or hPSCs are differentiated along the neural lineage [42]. The noteworthy temporal sequence of neuronal and glial differentiation corresponds to the timeline observed from limited samples of fetal tissues [42]. 

It is suggested that the intrinsic program governing neuronal and glial lineage development is retrained *in vitro* and highlights the feasibility of obtaining specific neuronal subtypes from hPSCs when appropriate inducers are applied at an optimal time point. In addition to extrinsic factors, forced expression of specific transcription factors, for example, *NGN2* and PAX6, could speed and enrich the generation of neural lineage from hPSCs [43–45]. However, this transcription factor-based approach is needed for the development of an exogenous DNA-free system prior to translating it to patients.

## 3. Human Pluripotent Stem Cells Accelerate Biomedical Research for Neurological Disorders

Understanding the molecular interactions underlying developmental disorders of the brain is hindered by limited accessibility to early embryos and an inadequate amount of stage- and cell type-specific materials. Although these developmental diseases are unlikely to be cured by cell replacement therapies, a complete picture of disease pathology by disease modeling will facilitate the discovery of effective compounds that can improve patient conditions [46]. Recent results indicate that the differentiation of hPSCs in culture follows the hierarchical set of signals that regulate embryonic development in the generation of the germ layers and specific cell types [47]. Establishment of *in vitro* differentiation models that recapitulate brain development will form the foundation for dissecting molecular interactions. The ability to access and manipulate populations representing early neural developmental stages in the hPSC differentiation cultures provides a new approach for addressing the questions of lineage commitment. 

The investigation of neural induction paradigms in hPSCs has significant implications for the insights into early human brain development. In recent years, more sophisticated and chemically defined culture systems have been developed. Anti-BMP signaling is thought to play a crucial role in neural induction [12, 31]. Further studies found that the high efficiency of neural induction with BMP antagonist treatment is consistent with its role in the default model of neural induction [48]. In addition to the shared signaling pathways, temporal consequences are similar between *in vivo* and *in vitro* systems. For example, during *in vivo* development, the neural tube formation completes when the human embryo is approximately 3 weeks old. On the other hand, *in vitro *differentiation of hPSCs toward neural lineage is characterized by the formation of a neural rosette observed at about days 15–17 of differentiation [12, 17], reminiscent of the transverse-section of the neural tube. It can be speculated that *in vivo* development events in terms of spatial and temporal changes are grossly recapitulated during the *in vitro *formation of neural rosettes. One of the characteristic features of neural progenitor cells is the positional identity they acquire during neural induction and patterning, which plays a key role in the fate specification of neuronal subtypes. The positional information is imparted upon neural progenitor cells via morphogenetic gradients secreted by surrounding tissues. Partially, to mimic the positional information in a culture petri dish, morphogens that affect rostrocaudal and dorsoventral fate choices are applied together or in sequence. Applying FGF8, which influences midhindbrain fate, and Shh, a ventralizing molecule, further induces hPSC-derived neuroepithelial cells into midbrain dopaminergic neurons [49]. On the other hand, the inhibition of Wnt signaling, together with the activation of SHH signaling, enhances forebrain induction from hPSCs [50]. Absence or presence of these positional morphogens in the* in vitro* differentiation system leads to the production of a variety of neuronal subtypes. This would mean that the addition of morphogens or small molecules at a specific time and space is needed to pattern neural progenitor cells into a particular neuronal subtype, and the foundation of this knowledge is originally based on the current understanding of neurodevelopment. The refinement of signaling pathways that control specific neuronal subtype specification *in vitro* will lay the foundation for studying affected neuron pathology in human neurological diseases.

In addition to modeling early brain development, iPSCs are a powerful tool for modeling diseases [46]. The idea of disease modeling is to derive iPSCs from the patient's own cells and then induce them into the pathogenic cell types* in vitro*. Several laboratories have already established hiPSCs from patients suffering from Parkinson's disease, amyotrophic lateral sclerosis, and spinal muscular dystrophy [51, 52]. Upcoming sophisticated differentiation and purification protocols would be necessary to generate cells that show comparable physiological conditions to disease stages. Moreover, hPSCs and their differentiated derivatives could be applied to chemical compound screening assays for the development of new potential pharmaceuticals and toxic or mutagenic reagents [53]. While primary cell cultures or established cell lines are commonly used for both purposes, hPSCs offer several advantages. The developmental equivalence of hPSC-derived and embryonic populations provide a more rigorous system for evaluating the teratogenic and embryotoxic effects of a substance, in addition to general mutagenic and cytotoxic effects [54]. A protocol based on the differentiation of hPSCs has been established and validated for use in toxicity testing [55]. Additionally, genetic modification enables the tailoring of hPSC lines for specific purposes. For example, specific genes can be altered to increase sensitivity to mutagens or drugs [56, 57], or tissue-specific reporter genes can be introduced to detect changes in gene expression induced by toxic chemicals or therapeutic agents [58]. 

## 4. Therapeutic Promises of Human Pluripotent Stem Cells for Neurological Disorders: Perspectives of Animal Studies 

Neurological disorders are the complex disintegration of neurons as well as many types of neuroglia in the brain and/or spinal cord. The early phase therapy, such as applying trypsin plasminogen activators (tPAs) in stroke patients during the first few hours, can be used only in early diagnosed patients [59]. Nevertheless, there is still no effective treatment, which can ameliorate that the functional deficit exists in the human subjects. Several researchers established the protocols to generate neural progenitor/stem cells, motor neurons, oligodendrocyte progenitor cells *in vitro* and then transplanted these cells into various animal models in order to verify the ability to restore neuronal functions *in vivo*. There are promising evidences of differentiation, maturation, and integration of the grafted cells into the endogenous neural circuitry in animal models [60, 61]. The hPSC-derived cells, which were introduced into the animal models, were restricted to one specific cell lineage in order to reduce the risk of tumorigenesis when compared with the direct transplantation of hPSCs [62]. Experimental studies in animal models are necessary to extrapolate the therapeutic effects of transplanted cells. Currently, there are two main strategies for assessing the efficacy of hPSCs for treating neurological disorders in animal models. First, hPSC-derived neurons and/or neuroglia were substituted into mice with injured neural circuitry by using intracerebral transplantation. Second, hPSCs were delivered systemically or locally into the brain where they might act through some other mechanisms to promote the differentiation, such as immunomodulation, neuroprotection, and stimulation of angiogenesis. 

The neural derivatives of hPSCs are plausible sources for cell replacement therapies. Several diseases were already experimented in an animal setting, such as stroke and brain ischemia [63–66], spinal cord injury [23, 67, 68], Parkinson's disease [69], spinal muscular atrophy, amyotrophic lateral sclerosis [24, 70], and demyelinating diseases [71]. These provisional studies were performed in specific diseases environment in order to endow a prospect of pathological conditions, in which the results could be implemented for clinical interpretations. In this section, we discuss some prominent examples of neurological disorders that have been conducted in animal transplantation studies using hPSCs.

### 4.1. Stroke

The vast majority of neurological disorders falls into the group of devastating pathology and takes place in the cerebral arteries which are called *cerebrovascular accident* (CVA) or *stroke *[72]. Stroke is an overwhelming condition with lifelong functional deficits in patients due to tremendous loss of neuronal circuitry in the brain. The recovery of stroke patients is often incomplete even when they have received physical therapy training to promote functional recovery. Consequently, there is great enthusiasm for using cell therapy to restore and replenish dead cells and tissues after brain injury an expectation of functional improvements. Stroke is typically a consequence of a thrombotic or embolic occlusion in a major cerebral artery, most often the middle cerebral artery (MCA). Experimental focal cerebral ischemia models have been established to imitate human stroke and serve as an indispensable tool in the field of stroke research [73]. Models of cerebral ischemia can either be artery or vein occlusion via mechanics or thromboembolisms. An ischemic model is categorized into global and focal ischemia. Global ischemia is the restriction of blood flow, affecting the entire brain area, whereas focal ischemia is characterized by a reduction of cerebral blood flow in a distinct region of the brain. The global ischemia can be further divided into complete and incomplete types, while focal ischemia can be performed in both focal and multifocal cerebral ischemia [74]. Moreover, cerebral vessel occlusion can occur either in the proximal middle cerebral artery (pMCAo) (large vessel occlusion) or distal MCA (dMCAo) (small vessel occlusion). Thrombotic occlusion can be induced either via the injection of blood clots or thrombin into the MCA or by photo-thrombosis after intravenous injection of Rose Bengal [75]. Recently, there has been evidence in hPSCs transplantation, which focused on cortical injury. The studies were performed by dMCAo rather than transient MCAo models, which turned to both cortical and striatal injury [63, 64]. These studies showed a substantially decreased infarct volume after the transplantation of neural progenitor cells derived from hPSCs. Nevertheless, until now, experimental stroke studies in transgenic animal models have had limited success. This highlights the significant contribution of vascular risk factors found in certain clinical situations, which is also viable in the human systems [75, 76]. 

### 4.2. Spinal Cord Injury

Spinal cord injury (SCI) is another important neurological disorder which can be used to reveal the therapeutic effects of transplanted cells in animal studies. SCI causes permanent paralysis in patients due to the low rate of regeneration in the central nervous system (CNS). Robust cell death in the injured region happens from seconds to weeks after SCI, which results in the formation of the cavities or cysts that block the ascending and descending neurotransmission. This phenomenon occurs immediately after SCI, including neuronal fiber damage, mass ischemic neural cell necrosis and apoptosis, and glial scar formation, and leads to extensive secondary tissue injuries. SCI is a devastating condition, characterized by the disruption of axonal connections, failure of axonal regeneration, and loss of motor and sensory function. The therapeutic promise of stem cells has been focused on cell replacement, but many obstacles remain, in particular neuronal integration following transplantation into the injured CNS. Various cell types have been selected based on their ability to form myelin protein, promote and guide axonal growth, and bridge the site of injury. In addition, transplanted cells also secrete trophic factors, which may have neuroprotective effects and/or promote plasticity in the spared spinal cord. Therefore, the advantageous effects of these cellular therapies are multifactorial and often difficult to attribute to one single mechanism. Most cell transplantations are delivered directly into the site of injury or adjacent area by injecting a few microliters of cell suspension via fine needles or glass capillaries. Attempts have been made to deliver cell substrates to the injured cord via intrathecal injection [77–79] or even systemically via intravenous infusions. Rodent models of SCI are used, and the transplantation is typically performed 1-2 weeks after the injury and referred to “subacute” treatment, since transplantations performed immediately after “acute” injury generally yield poor results due to the robust inflammatory response initiated at the time of injury [80]. In order to promote functional recovery, stem cell transplantation must be done after inflammatory responses. The optimal time-point for cell therapy contains several benefits, for instance, the inhibition of neuronal apoptosis and necrosis, the enhancement of neuronal regeneration, and the promotion of axon regeneration and remyelination; therefore, understanding the timeline of secondary damage cascades is definitely critical [81]. Reportedly, hPSCs have been investigated and their therapeutic efficacy and safety for SCI* in vivo* have been verified [23, 68]. hESCs were differentiated into motor neuron progenitors (MPs) and oligodendrocyte progenitor cells (OPCs). The functional recovery was compared after transplanted either MPs or OPC alone, or the combination of MPs and OPCs into the SCI mice. The functional locomotor recovery of transplanted animals with MPs and OPCs showed significant improvement of the hind limb, better than the groups that were treated with a single cell type [23]. A protocol was recently developed for the creation of long-term self-renewing neuroepithelial-like stem (lt-NES) cells from hPSCs [68]. These hPSC-lt-NES cells exhibit reliable characteristics, including homogenous population, continuous expandability, stable neuronal/glial differentiation ability, and the capacity to generate functional mature neurons in a monolayer platform [68]. Promisingly, when transplanted into the SCI model (NOD-SCID mice), these transplanted cells have a comparable therapeutic potential, similar to neural stem cells (NSCs) derived from human fetal spinal cord (hsp-NSCs). 

### 4.3. Parkinson's Disease

The key pathology of Parkinson's disease (PD) is motor symptoms. This is due to the progressive degeneration of mesencephalic dopaminergic (DA) neurons that project to the striatum and subsequent reductions in striatal dopamine levels. Initial pharmacological treatment with L-dihydroxyphenylalanine (L-DOPA) can ameliorate symptoms, but effectiveness of this compound gradually decreases overtime. The progression of motor deficits then requires additional treatments, including deep brain stimulation. The breakthrough lines of evidence of promising stem cell research are appealing as an alternative choice to fight against the disease. In the laboratory, PD animals can be induced by systemic injection of 1-methyl-4-phenyl-1,2,3,6-tetrahydropyridine (MPTP) [69] or the central administration of the neurotoxin, 6-hydroxydopamine injection [82]. MPTP treatment is currently a gold standard for generating PD animal models. Ingestion of this compound results in the fabrication of motor deficits that are similar to PD. MPTP subsequently converts to MPP^+^ in the brain, which is later reuptaken into DA neurons by the dopamine transporter (DAT). MPP^+^ exerts a blockage on the electron transport chain in the mitochondria of DA neurons and generates reactive oxygen species (ROS), which effectively kill the neurons [83]. There is still controversy as to whether MPTP-treated mice contained Lewy body-like inclusions, a hallmark of PD. Only a few inclusions were found after 3 weeks of chronic MPTP treatment. By 24 weeks, several of the remaining tyrosine hydroxylase (TH) positive neurons contained *α*-synuclein and ubiquitin-immunoreactive inclusions [84]. On the other hand, 6-hydroxydopamine (6-OHDA) is preferentially transported into DA neurons by the DAT, where it gets accumulated and produces ROS. This toxin is needed for the administration via intraparenchymal injection because it does not cross the blood-brain barrier. Unilateral administration results in asymmetric circling behaviour, which is suitable for the evaluation of therapeutic interventions [85]. The preclinical study using MPTP-treated monkey models showed the therapeutic effects of transplanted cells at different stages, undifferentiated hESCs, and hESC-derived neural progenitor cells. As expected, the transplantation of undifferentiated hESCs at day 14 (before SHH and FGF8 induction) showed tumor formation. To induce neural progenitor cells for the transplantation, hESCs were induced with SHH for either 35 or 42 days prior to the addition of FGF8, BDNF, and GDNF for another 1-2 weeks. The grafts were well demarcated and showed no malignancy or even teratoma. The transplanted cells were mainly contributed to immature neural cells and DA neurons. The evaluation of functional neurological scores revealed that monkeys implanted with day 42 neurospheres had significant behavioural improvement over another group. The key statement of this finding was to support a prolonged differentiation maturation of neural progenitor cells that led to a favourable result regarding reduced tumor growth and functional grafts [69]. 

### 4.4. Motor Neuron Diseases

Recent progress in cell-based modeling using hPSC-derived motor neurons (MNs) has opened a new window to understanding the pathological development of motor neuron diseases. MNs exclusively reside within the ventral horn of the spinal cord and project axons to muscles to control their activity in organized and discrete patterns as the lowest unit in the hierarchy of the motor pathway. The most remarkable MN diseases are spinal muscular atrophy (SMA) and amyotrophic lateral sclerosis (ALS). SMA is characterized by severe muscle weakness, symmetrical proximal muscle weakness, lack of motor development, and hypotonia [86]. SMA is the cascade of genetic deficits, resulting in the decrease of survival of motor neuron (SMN) protein levels. The transgenic model of SMN gene deletion has yielded important insights into the pathogenesis of the disease. ALS, also known as Lou Gehrig's disease, is a devastating adult-onset neurodegenerative disorder, characterized by a progressive loss of both cortical and spinal motor neurons. The clinical manifestations of ALS are progressive myasthenia and general amyotrophy, eventually resulting in paralysis and death [87]. Transgenic mice that overexpress mutant human superoxide dismutase 1 (*SOD1*) gene reproduce clinical and histopathological features of human ALS. This animal model is of interest for the investigation of ALS pathogenesis and for the testing of therapeutic approaches [88]. Stem cell-based therapies have demonstrated therapeutic potential in SMA and ALS. For example, MNs derived from hiPSCs, which were obtained from an SMA patient, exhibited shortened neurite extensions and diminished survival in culture compared to healthy MNs [89]. Conversely, ALS patient-derived MNs did not present any defect [52]. MNs derived from hESCs proved their therapeutic capacity via *in ovo* and *in vivo* transplantation of the spinal cord [24]. The results revealed that MNs can survive for at least 6 weeks in the rat spinal cord and also outgrow the axons toward peripheral targets. This study provided strong promise for future applications in preclinical models and translational applications of hESC-derived MNs. It will be essential to develop animal models for specific conditions of MN diseases to address the question of whether hESC-derived MNs can survive and function in a particular MN disease environment.

### 4.5. Multiple Sclerosis

Multiple sclerosis (MS) is an autoimmune-mediated inflammatory disease. It is characterized by multifocal regions of inflammation and myelin devastation, which leads to demyelination and neuronal loss. MS is presented by multiple signs and symptoms, with relapses and remissions of the disease stages. Even though there are several approved treatments for MS, many patients do not optimally respond to those approaches. A number of laboratory animals are described as demyelinating disease models. Experimental autoimmune encephalomyelitis (EAE) is one of the most commonly characterized disease and is employed as an animal model for MS [90]. EAE mice are a model of CNS autoimmune disease that follows immunization with certain CNS antigens and subsequent administration of heat-inactivated Mycobacterium tuberculosis and pertussis toxin. Induced animals will develop a strong immune response with signs of inflammation, demyelination, axonal loss, and gliosis, which is similar to MS pathology in humans. Besides EAE, it is previously reported that an MS model could be induced by viral infection [71]. A number of human viral pathogens have been considered to be involved in eliciting myelin-reactive lymphocytes and/or antibodies that subsequently infiltrate the CNS and damage the myelin sheath [91, 92]. Mouse hepatitis virus (MHV) infection results in an acute encephalomyelitis, followed by chronic demyelination in animals. This observation is similar to clinical and histologic profiles of MS patients. Human oligodendrocyte progenitor cells (OPCs) derived from hESCs transplanted in the MS model have been shown to promote remyelination in mice that are persistently infected with MHV [71]. The ability of preclinically applicable cells to facilitate remyelination in an animal model of MS would be a crucial step towards developing novel therapies.

## 5. Safety Considerations of Using hPSC-Derived Neurons

As mentioned, hPSCs offer a possible unlimited supply of disease-specific progenitor cells for regenerative medicine. The selected cell types can be variable, according to material sources, culture conditions, and differentiation protocols. These issues are important and need to be considered prior to translating preclinical outcomes into clinical studies. Undefined biological supplements, which are used for cell sustenance in the processes of isolation, expansion, and differentiation of hPSCs, may cause undesired problems in patients. For example, the maintenance of the hPSCs in growth-arrested mouse embryonic fibroblasts (MEFs) may worsen therapeutic potential due to the transmission of xenopathogens, altering the genetic background, and promoting the expression of immunogenic proteins in hPSCs [93]. Recently, there has been evidence of using xenofree iPSC-derived neural progenitor cells transplanted into ischemic stroke models [65]. This study represented the success of the derivation iPSCs in feeder- and serum-free systems. The cells could still be differentiated into functional neurons, which are transplantable into stroke animals. 

Another challenge for the clinical considerations of hPSCs is how to efficiently induce and enrich pluripotent cells for a desired phenotype. In order to select desired cell types, certain approaches have been applied, such as antibody-based selection for specific surface antigens by FACS sorting. However, these approaches need to be improved in order to produce a large and viable population. To avoid tumor formation in the engrafted tissues, the pluripotent state of differentiated hPSCs needs to be verified to confirm no existing contamination of pluripotent cells among differentiated cells. Directing the pluripotent cells into the multipotent NSCs appears to be necessary for safety considerations and effectiveness in clinical translation. The precise stages of differentiation for transplantation remain unclear. The gene and epigenetic profiles are needed to validate reliable cell types before transplantation [94, 95]. Therefore, safe and effective clinical translation of hPSCs for neurological disorders is required for thoroughly understanding of both inherent and noninherent cellular mechanisms that maintain pluripotency and differentiation programs. 

## 6. Future Challenges of Neural Derivatives for Biomedical Research and Clinical Applications

Based on the principle of developmental biology, a set of neurons and glia has been successfully differentiated from hPSCs. Modeling neurological diseases using hPSCs has the potential to provide a valuable impact on biomedical research and regenerative medicine. The possible risks are that the high variability in the protocols generates specific neuron subtypes and the ability to mimic disease-specific phenotypes. Manipulation of culture environment, such as oxidative stress, may enhance pathological phenotypes of neural derivatives derived from diseased iPSCs [96]. Although hPSC-derived neural derivatives were proved to be functional *in vitro* and were able to correct phenotypes of diseased mice, there are still several issues remaining to be solved prior to realizing clinical translation, for example, the purity of transplanting cells, sites of transplantation, graft versus host diseases, tumorigenesis, and integration of transplanted cells. The development of safer iPSCs is necessary to avoid exogenous DNA integration that can subsequently affect genomic alterations and cause tumor. Finding specific biomarkers for cell sorting may provide a solution to the selection of desired cell types for transplantation. In principle, patient-specific iPSCs should provide immunogenically matched tissues; however, further validation is still needed for safety issues in order to avoid any possible tissue rejections [97, 98]. 

Another key advantage of hiPSCs over the current transplantation approaches is the possibility of correcting mutations by homologous recombination technology. The generation of genetically corrected iPSCs by genome-editing technology is a mainstay for the advancement of iPSC technology to generate healthy iPSC lines for individual patients [99, 100]. The differentiation of specific neural derivatives from genetically corrected iPSCs could provide a source of neurons for therapeutic transplantation. Besides, several neurological disorders are noncell autonomous, and neuronal death is driven by factors in the cellular environment, such as oxidative stress and inflammatory cytokines. The transplantation of nonneuronal cells, for example, astrocytes and oligodendrocytes, to refine the microenvironment conditions is thus a practical strategy. Another interesting concept in stem cell therapy is the dual effects of stem cell transplantation together with noninherent effects, such as rehabilitation or exercise. Since the majority of neurological disorder patients receive physiotherapy training, the combination of two approaches could enhance the therapeutic outcomes. To this end, the concerted efforts on hPSC research have made great progress toward cell replacement therapies; however, it is important to support the most carefully designed clinical studies for the best safety for patients in the future.
